# SIP1 is a downstream effector of GADD45G in senescence induction and growth inhibition of liver tumor cells

**DOI:** 10.18632/oncotarget.5602

**Published:** 2015-09-10

**Authors:** Guiqin Xu, Li Zhang, Aihui Ma, Yu Qian, Qi Ding, Yun Liu, Boshi Wang, Zhaojuan Yang, Yongzhong Liu

**Affiliations:** ^1^ State Key Laboratory of Oncogenes and Related Genes, Shanghai Cancer Institute, Renji Hospital, Shanghai Jiaotong University School of Medicine, Shanghai, China

**Keywords:** GADD45G, SIP1, cell senescence, hepatocellular carcinoma

## Abstract

Cellular senescence evasion caused by the inactivation of tumor suppressive programs is implicated in tumor initiation and therapeutic resistance. Our previous study has shown that the downregulation of growth arrest and DNA damage 45G (GADD45G) contributes to senescence bypass in hepatocellular carcinoma (HCC). Here, we report that the Smad-interacting protein-1 (SIP1) is transcriptionally activated and functions critically in the GADD45G-induced tumor cell senescence. Knockdown of SIP1 significantly abrogates the suppressive effects of GADD45G on the growth of xenografted liver tumor *in vivo*. The essential role of SIP1 in GADD45G activities is further validated in the model of the proteasome inhibitor MG132-induced cell senescence. We further show that JNK but not p38 MAPK activation is involved in the GADD45G-mediated SIP1 upregulation, and that JNK inhibition counteracts the GADD45G-induced cellular senescence. More importantly, we show that GADD45G and SIP1 expression are coincidently downregulated in primary human HCC tissues. Together, our results establish that the dowregulation of GADD45G-SIP1 axis may contribute to cellular senescence evasion and HCC development.

## INTRODUCTION

Hepatocellular carcinoma (HCC), a highly lethal cancer, is one of the most common tumors worldwide. HCC development is usually associated with liver cirrhosis and chronic hepatitis B virus (HBV) and hepatitis C virus (HCV) infections [1]. Emerging evidence has shown that in order to acquire the peculiarity of cellular immortality, human tumors frequently exhibit loss of p53 and p16^INK4a^-Rb-medieated senescence program [2, 3]. Similarly, HCC tumor cells are also believed to bypass hepatocellular senescence to gain immortality [4]. Indeed, HCC is one of the major tumors displaying p53 mutations and p16^INK4a^ inactivation [5]. Downregulation of p16 via DNA methylation is responsible for Hepatitis C virus Core protein to overcome stress-induced premature senescence in HCC [6]. Studies have shown that senescence process can be reactivated in HCC cells if the protumorigenic events are inactivated. For instance, the suppression of *c-myc* oncogene results in tumor regression by inducing premature senescence in murine HCCs [7]. miR-34a regulates cellular senescence through the modulation of telomere pathway by targeting the c-Myc and FoxM1 gene in HCC tissues and cell lines [8]. In addition to the functional defeats in the classical cell cycle check-points, such as p53, retinoblastoma (Rb), and INK4a-ARF, the dys-regulation of nonclassical senescence inducers, as an independent or a complementary mechanism, may be also required for senescence evasion and efficient tumorigenesis [9–12].

Cell senescence, a physiological program of terminal growth arrest in response to alterations of telomeres or different forms of stress, is considered to be a strong anticancer mechanism [13, 14]. Unlike reversible cell cycle arrest (quiescence), a characteristic of senescent cells is loss of proliferative potential, while the quiescent cells still have the ability to restart proliferation [15]. There is a terminology named geroconversion for the transition of proliferative arrest to irreversible senescence. This process can be driven by growth-promoting pathways such as mTOR pathway and suppressed by Rapamycin [16, 17].

Growth arrest and DNA damage 45G (GADD45G) encodes a stress-responsive protein that is involved in DNA damage response and cell growth arrest through modulating a number of cellular proteins, including the proliferating cell nuclear antigen (PCNA), p21, Cdk1, cdc2/cyclin B1, p38 and c-Jun N-terminal kinase (JNK) [18–21]. Moreover, GADD45G levels are remarkably downregulated in the different types of solid tumors compared to their corresponding normal tissues [22]. We have previous shown that GADD45G can robustly elicit HCC cell senescence independently of the functional presence of p53, p16^INK4a^ and Rb, and that GADD45G downregulation may contribute to senescence bypass and promote tumor growth in the development of HCC cells [23]. However, the mechanisms underlying GADD45G-mediated antitumor activity are not well understood. In this study, we show that the Smad-interacting protein-1 (SIP1) is a key effector downstream of GADD45G in programming cellular senescence in liver tumor cells. The coincident downregulation of GADD45G and SIP1 in clinical HCCs further indicates the pathological relevance of the deregulation of GADD45G-SIP1 axis in the development of HCC.

## RESULTS

### SIP1 is required for GADD45G-induced tumor cell senescence

**Figure 1:**
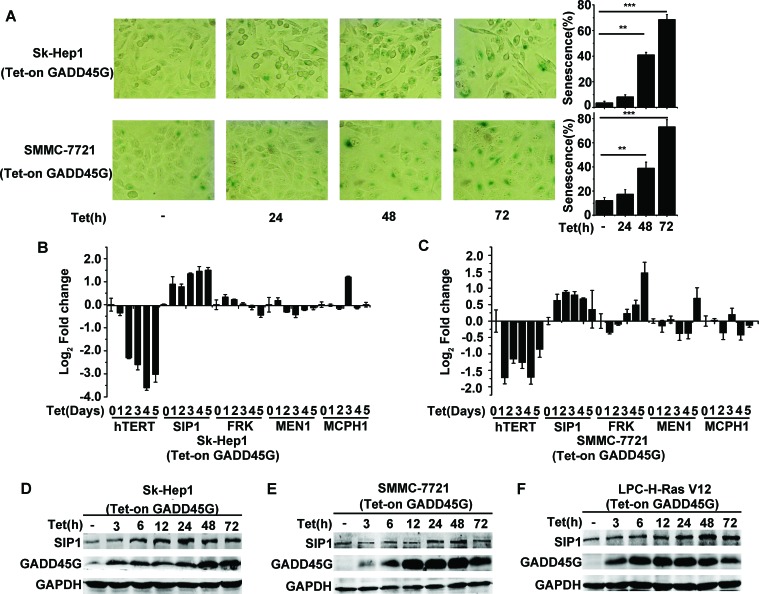
SIP1 activation in GADD45G-induced tumor cell senescence **(A)** Tet-on-GADD45G-Sk-Hep1 and Tet-on-GADD45G-SMMC-7721 cells were cultured for the indicated times in the presence of 2.5μg/mL DOX (Tet-on) for GADD45G induction. The representative images of SA-β-gal staining (left panel) and the percentage of SA-β-gal positive cells (right panel) are shown. Data shown are mean ± SD from three independent experiments (**P < 0.01, ***P < 0.001). SD, standard deviation. **(B-C)** Tet-on-GADD45G-Sk-Hep1 **(B)** and Tet-on-GADD45G-SMMC-7721 **(C)** cells were cultured with DOX for the indicated times. Cell lysates were harvested for analysis of indicated genes by qRT-PCR. **(D-E)** Tet-on-GADD45G-Sk-Hep1 **(D)** and Tet-on-GADD45G-SMMC-7721 cells **(E)** were cultured with DOX for the indicated times. Cell lysates were analyzed for SIP1 expression by Western blot. **(F)** Tet-on-GADD45G-H-Ras V12-transformed mouse liver progenitor cells (Tet-on-GADD45G-LPC-H-Ras V12) were cultured with DOX for the indicated times. SIP1 levels were analyzed by Western blot.

In agreement with previous observation, we confirmed that the induction of GADD45G expression in Sk-Hep1 cells (Tet-on-GADD45G-Sk-Hep1) and SMMC-7721 cells (Tet-on-GADD45G-SMMC-7721) resulted in characteristic morphological changes common in senescent cells and a dramatic increase in the proportion of SA-β-Gal-staining positive cells (Figure 1A). Since GADD45G-induced senescence is related to the repression of hTERT, we therefore examined the change pattern of the hTERT upstream regulatory genes, including SIP1, FRK, MEN1, MCPH1 [24], in Sk-Hep1 and SMMC-7721 cells with or without GADD45G induction. As expected, GADD45G induction significantly inhibited the expression of hTERT mRNA. Surprisingly, we found that the levels of SIP1 mRNA were significantly elevated in the cells upon GADD45G induction (Figure 1B, 1C). The change in SIP1 mRNA expression was also reflected at the protein levels in Sk-Hep1 and SMMC-7721 cells upon GADD45G induction (Figure 1D, 1E). In addition, GADD45G-induction of SIP1 expression similarly occurred in H-Ras V12-transformed mouse p53−/− liver progenitor cells (LPC-H-Ras V12 cells) (Figure 1F).

**Figure 2:**
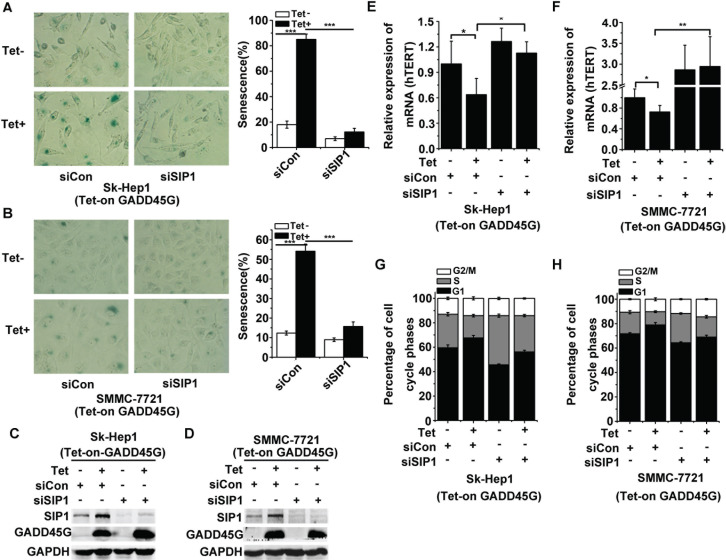
SIP1 inhibition attenuates GADD45G-induced tumor cell senescence *in vitro* **(A-B)** Tet-on-GADD45G-Sk-Hep1 **A.** and Tet-on-GADD45G-SMMC-7721 cells **(B)** were transfected with siRNA targeting SIP1 (siSIP1) or with control siRNA ( siCon), then cultured for 3 days with or without GADD45G induction. Representative images of SA-β-gal staining (left panel) and the percentages of positive cells (right panel) are shown. Data shown are mean ± SD from three independent experiments (***P < 0.001). SD, standard deviation. **(C-D)** Tet-on-GADD45G-Sk-Hep1 **(C)** and Tet-on-GADD45G-SMMC-7721 cells **(D)** were transfected with the indicated siRNAs, then cultured for 3 days with or without GADD45G induction. The efficiency of the siRNA for the downregulation of SIP1 protein was confirmed by Western blot. **(E-F)** hTERT mRNA levels were measured in Tet-on-GADD45G-Sk-Hep1 **(E)** and Tet-on-GADD45G-SMMC-7721 **(F)** cells with the indicated treatments. Data shown are mean ± SD from three independent experiments (*P < 0.05, **P < 0.01). SD, standard deviation. **(G-H)** Tet-on-GADD45G-Sk-Hep1 **(G)** and Tet-on-GADD45G-SMMC-7721 cells **(H)** with the indicated treatments were harvested for analysis of the percentage of cells in each cell cycle phase by fluorescence-activated cell sorting. Data shown are mean ± SD from three independent experiments. SD, standard deviation.

Previous work has revealed the roles of SIP1 in negative regulation of hTERT and in reprogramming replicative senescence in p53- and p16^INK4a^-dificient HCC cells [25]. Therefore, we raised the question of whether SIP1 induction is critical for GADD45G-induced tumor cell senescence. We treated Sk-Hep1 and SMMC-7721 cells with siRNA targeting SIP1 or the control siRNA for 24 hours and then cultured these cells with or without DOX (Tet-on) for GADD45G induction. At 72 hours after DOX treatment, we found that SIP1 knockdown efficiently counteracted GADD45G-induced senescence, as scored by the percentage of SA-β-gal-positive cells (Figure 2A, 2B). The efficiency of the siRNA for inhibiting SIP1 expression in cells was confirmed by Western blot analysis (Figure 2C, 2D). Meanwhile, we detected whether the SIP1 downregulation was able to prevent GADD45G-mediated inhibition of hTERT expression. Indeed, the decrease in hTERT expression in the cells with GADD45G induction was blocked by SIP1 inhibition (Figure 2E, 2F). Consistently, the results of cell-cycle analyses demonstrated that SIP1 knockdown by siRNA efficiently attenuated GADD45G-induced G1 arrest (Figure 2G, 2H). These results collectively indicate that SIP1 plays an important role in GADD45G-induced cell senescence.

### GADD45G and SIP1 function in the proteasome inhibitor MG132-induced tumor cell senescence

**Figure 3:**
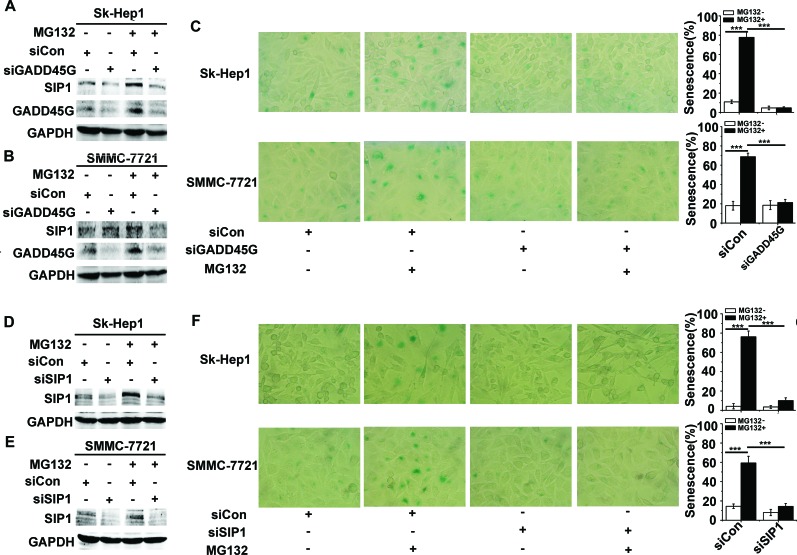
GADD45G and SIP1 are required for the proteasome inhibitor MG132-induced tumor cell senescence **(A-B)** Sk-Hep1 **(A)** and SMMC-7721 **(B)** cells were transfected with siRNA targeting GADD45G or a control siRNA for 24 hours, then treated with or without 3 μM MG132 for 2 hours. Cells were harvested at 24 hours after MG132 treatment. Western blot analysis shows levels of SIP1 and GADD45G proteins. **(C)** Sk-Hep1 and SMMC-7721 cells were treated as indicated above. SA-β-gal staining analysis was performed for senescent cells. Data shown are mean ± SD from three independent experiments (***P < 0.001). SD, standard deviation. **(D-E)** Western blot analysis of SIP1 levels in Sk-Hep1 **(D)** and SMMC-7721 cells **(E)** which were transfected with the SIP1 siRNA or a control siRNA, then cultured for 24 hours with or without pretreatment of MG132. **(F)** Sk-Hep1 and SMMC-7721 cells were transfected with the indicated siRNAs, then cultured for 24 hours with or without pretreatment of MG132. Representative images showing SA-β-gal staining (left panel) and the percentage of positive cells (right panel) are shown. Data shown are mean ± SD from three independent experiments (***P < 0.001). SD, standard deviation].

Considering that GADD45 family proteins act as key players in cellular stress responses, we examined whether endogenous GADD45G-SIP1 activation contribute to stresses-triggered cell senescence. We employed a model of the drug MG132-induced cell senescence, in which process the essential role of GADD45G has been demonstrated [23]. As shown in Figure 3A, 3B, transient treatment of Sk-Hep1 and SMMC-7721 with 3 μM MG132 for 2 hours, significantly increased levels of SIP1 and GADD45G were displayed; however, in the cells pretreated with siRNA specific for GADD45G, levels of SIP1 were accordingly downregulated. As expected, at 24 hours after MG132 treatment, approximately 60%- 80% of these cells were stained positively for SA-β-gal activity, whereas knockdown of GADD45G by siRNA markedly decreased the percentages of SA-β-gal-positive cells (Figure 3C). Intriguingly, in the cells pretreated with siRNA for the downregulation of SIP1, MG132-induced cell senescence was profoundly abrogated (Figure 3D, 3E, 3F). These results suggest that the activation of GADD45G-SIP1 axis may play a significant role in certain drugs-mediated tumor cell growth arrest.

### JNK activation is required for GADD45G-induced SIP1 expression

**Figure 4:**
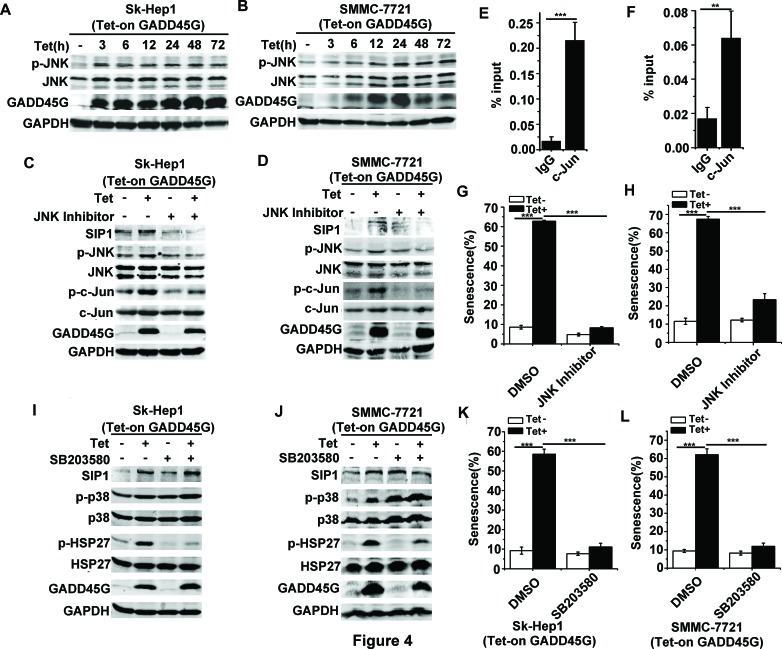
JNK but not p38 MAPK activation contributes to GADD45G-induced SIP1 upregulation **(A-B)** Tet-on-GADD45G-Sk-Hep1 **A.** and Tet-on-SMMC-7721 cells **B.** were cultured with DOX for the indicated times. p-JNK levels were examined by Western blot. **(C-D)** Tet-on-GADD45G-Sk-Hep1 **(C)** and Tet-on-GADD45G-SMMC-7721 cells **(D)** were treated with 10μM JNK Inhibitor II for 1 hour, then cultured in the presence or absence of DOX for GADD45G induction for another 24 hours. Western blot analysis shows the levels of the indicated proteins. **(E-F)** Tet-on-GADD45G-Sk-Hep1 **(E)** and Tet-on-GADD45G-SMMC-7721 **(F)** cells were cultured for 48 hours with GADD45G induction, then the cells were cross-linked, sonicated and subjected to ChIP with anti-c-Jun antibody beads or agarose-conjugated anti mouse IgG beads. Isolated DNA was employed in qPCR with SIP1 promoter-specific primers. Data shown are mean ± SD from three independent experiments (**P < 0.01, ***P < 0.001). SD, standard deviation. **(G-H)** Tet-on-GADD45G-Sk-Hep1 **(G)** and Tet-on-GADD45G-SMMC-7721 **(H)** cells were treated with 10μM JNK Inhibitor II for 24 hours, then washed and cultured for 72 hours with or without GADD45G induction. The percentage of SA-β-gal positive cells is shown. Data shown are mean ± SD from three independent experiments (***P < 0.001). SD, standard deviation. **(I-J)** Tet-on-GADD45G-Sk-Hep1 **(I)** and Tet-on-GADD45G-SMMC-7721 cells **(J)** were treated with 10μM SB203580 for 24 hours with or without GADD45G induction. Western blot analysis shows the levels of the indicated proteins. **(K-L)** The SA-β-gal assay was performed in Tet-on-GADD45G-Sk-Hep1 **(K)** and Tet-on-GADD45G-SMMC-7721 cells **(L)** treated with 10μM SB203580 for 72 hours with or without GADD45G induction. The percentage of SA-β-gal positive cells is shown. Data shown are mean ± SD from three independent experiments (***P < 0.001). SD, standard deviation.

We next investigated the mechanisms underlying SIP1 upregulation in GADD45G-induced cell senescence. Previous evidence has shown that GADD45 family proteins function as the stress sensors in cells through activating p38 MAPK and JNK pathways [26, 27]. We therefore analyzed whether GADD45G-mediated activation of these MAPK pathways was responsible for SIP1 upregulation. As shown in Figure 4A, 4B, after GADD45G induction for the indicated times, the phosphorylation of JNK was substantially enhanced in Sk-Hep1 and SMMC-7721 cells. To test whether JNK inhibition is sufficient to suppress the GADD45G-induced activation of SIP1, we treated Sk-Hep1 and SMMC-7721 cells with JNK Inhibitor II for 1 hour and cultured them for another 24 hours in the presence or absence of GADD45G induction. The results showed that JNK inhibition remarkably reduced the GADD45G-induced upregulation of SIP1. Meanwhile, the phosphorylation of c-Jun, a transcription factor specifically phosphorylated by JNK, increased after GADD45G induction and the level of p-c-Jun decreased with JNK inhibitor treatment (Figure 4C, 4D). c-Jun is a major component of activating protein-1 (AP-1), which is a dimeric transcription factor [28]. Based on previous studies, c-Jun is effective in regulating SIP1 transcription via interacting with the AP-1 binding sites within SIP1 locus [29–31]. We further performed ChIP assays to test whether c-Jun directly regulates SIP1 mRNA transcription in Sk-Hep1 and SMMC-7721 cells with GADD45G induction. It was found that c-Jun could efficiently bind to the AP-1 cis-element located about 2.5kb downstream of the transcription start site (TSS) in the SIP1 promoter (Figure 4E, 4F, and data not shown). Taken together, these data indicate that JNK/c-Jun pathway directly activates SIP1 transcription. In addition, the cellular senescence induced by GADD45G was profoundly reduced in cells with JNK inhibitor pretreatment (Figure 4G, 4H).

We next examined whether p38 MAPK activation could contribute to SIP1 expression and senescence induction by GADD45G. Indeed, we observed an increase in p38 MAPK activity in cells upon GADD45G induction, as revealed by the levels of p-p38 and the activation of its substrate HSP27. However, the treatment of the p38 MAPK inhibitor SB203580, which efficiency was confirmed by the decrease in levels of p-HSP27, did not decrease SIP1 expression in cells with or without GADD45G induction (Figure 4I, 4J). Intriguingly, SB203580 treatment significantly inhibited senescence induction as indicated by SA-β-gal staining in Sk-Hep1 and SMMC-7721 cells (Figure 4K, 4L). It seems likely that the involvement of p38 MAPK activation in GADD45G-induced senescence is independent of SIP1 induction. Collectively, these results indicate that it is JNK but not p38 MAPK activation that contributes to GADD45G-induced SIP1 expression.

### SIP1 inhibition abrogates GADD45G-mediated inhibition of tumor growth *in vivo*

**Figure 5:**
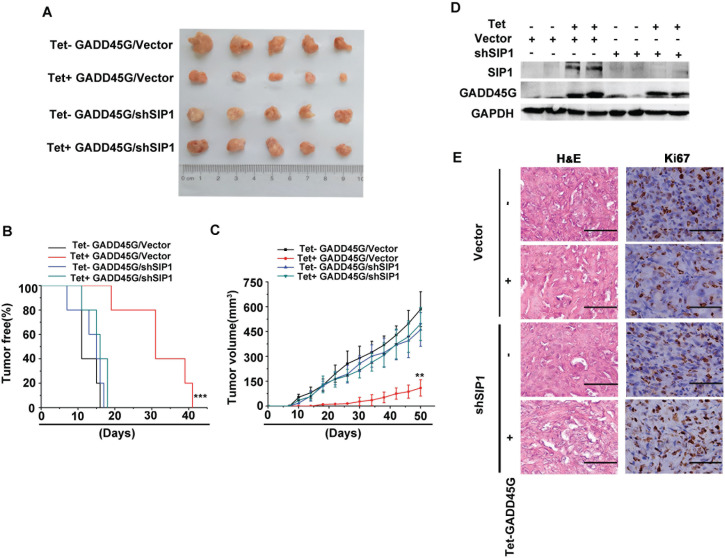
SIP1 inhibition attenuates GADD45G-mediated inhibition of tumor growth *in vivo* **(A)** Tet-on-GADD45G-Sk-Hep1/Vector and Tet-on-GADD45G-Sk-Hep1/shSIP1 cells were injected subcutaneously into nude mice (n = 5 for each group), and the mice were received normal water (Tet-) or the drinking water with 2mg/mL DOX (Tet+) after cell injection. The photograph presents the growth of the tumors in the mice with or without a 50-day treatment of DOX for GADD45G induction. **(B)** Kaplan-Meier's analysis of tumor onset. The group of Tet+ GADD45G/Vector mice compared with the control groups of mice (***P < 0.001). **(C)** Tumor volumes were measured at the indicated time intervals by measuring the maximum length and width of the tumor masses and were calculated by the following formula: V = length×(width)^2^/2 (**P < 0.01). **(D)** Successful induction of GADD45G and knockdown of SIP1 in the xenograft model were verified by Western blot. **(E)** Tumor sections from the mice were subjected to H&E staining and IHC against Ki-67. Original magnification, ×400. H&E, hematoxylin and eosin.

We next investigated the effects of SIP1 expression on the growth of liver tumor cells *in vivo*. Sk-Hep1 cells with Tet-on-GADD45G expression cassette were infected with lentiviruses carrying shRNA against SIP1 or the control shRNA, respectively. These cells were subcutaneously inoculated into nude mice and feed with normal water or DOX-containing water to induce GADD45G expression. All mice developed tumors at day 41 after tumor cell injection. As expected, the mice inoculated with tumor cells with GADD45G induction (Tet+ GADD45G/Vector) developed visible tumors much later than did the mice injected with the tumor cells without inducible GADD45G expression (Control: Tet− GADD45G/Vector). However, the average latency for tumor onset in the group with shRNA-mediated SIP1 inhibition and GADD45G induction (Tet+ GADD45G/shSIP1), was comparable to that in the groups without GADD45G expression, and much shorter than that in the group with GADD45G induction alone (Tet+ GADD45G/Vector ) (Figure 5A, 5B). In addition, the average volume of the tumors from the Tet+ GADD45G/shSIP1 group was significantly larger than those in the Tet+ GADD45G/Vector group (Figure 5A, 5C). Successful induction of GADD45G and knockdown of SIP1 in the xenograft model were verified by Western blot (Figure 5D). These results indicated that the GADD45G-induced inhibition of tumor growth was dependent on SIP1 upregulation. Moreover, the immunostaining results showed that, in the presence of GADD45G induction, the expression of the proliferation marker Ki-67 was robustly augmented in the tumor cells with SIP1 inhibition relative to those without SIP1 shRNA expression (Figure 5E). Taken together, these results clearly demonstrate that SIP1 downregulation abolishes the GADD45G-mediated inhibition of tumor growth *in vivo*.

### GADD45G and SIP1 expression levels are correlated in human hepatocellular carcinoma

**Figure 6:**
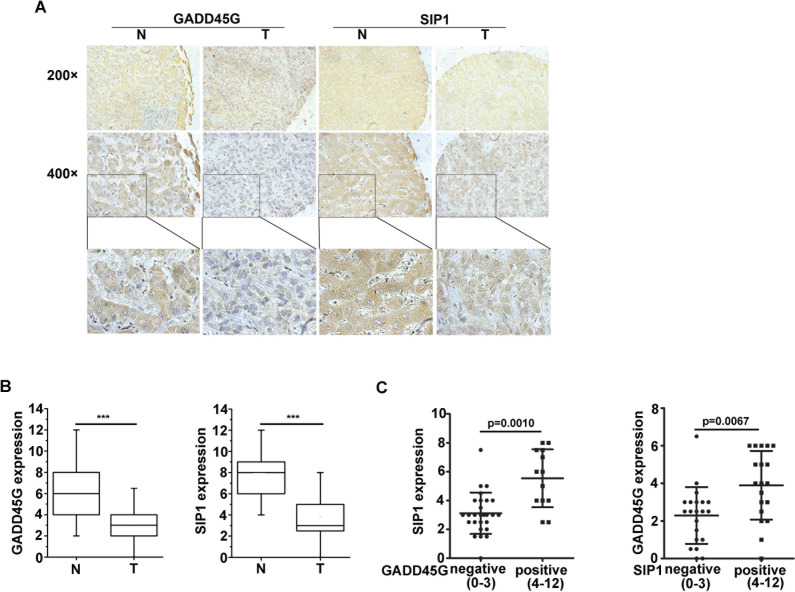
Expression levels of GADD45G and SIP1 were coincidently downregulated in human hepatocellular carcinoma **(A)** Representative images of GADD45G and SIP1 immunohistochemical staining. Original magnification, ×200 (upper panel); ×400 (middle panel). **(B)** Box-and-whisker plots of the staining of GADD45G and SIP1. The immunoreactive score is shown as mean ± SD (***P < 0.001). SD, standard deviation. **(C)** Statistical analysis of SIP1 expression in HCC sections negative (score, 0-3) and positive (score, 4-12) for GADD45G staining (left panel; *P* = 0.0010). SIP1-negative HCC sections exhibited a significantly lower GADD45G staining (right panel; *P* = 0.0067). The immunoreactive score is shown as mean ±SD. SD, standard deviation.

In order to investigate the correlation between GADD45G and SIP1 expression in human HCC samples, IHC staining of GADD45G and SIP1 was performed on 40 pairs of human liver tissue sections, including non-HCC and HCC tissues. We observed that both GADD45G and SIP1 expression were coincidently downregulated in the tissue sections from the same patient in HCC specimens compared to non-HCC tissues (Figure 6A, 6B). Box-and-whisker plots showed that the levels of GADD45G and SIP1 proteins were significantly lower in HCC sections than those in non-HCC tissues (*P* < 0.001) (Figure 6B). According to IHC scores of GADD45G and SIP1, approximately 72.5% (29 of 40) of adjacent nontumor regions showed GADD45G positivity (defined as score greater than 4), whereas only 9 of 40 (22.5%) HCC tissues were positive for GADD45G staining; similarly, positive staining of SIP1 was detected in approximately 92.5% (37 of 40) of nontumor sections and in 25% (10 of 40) of tumor areas (data not shown). Of note, there was a significant difference in SIP1 expression between GADD45G-negative and -positive tumors by statistical analysis (Figure 6C, left). When categorized with SIP1 expression in HCC tissues, the negative group had remarkably lower GADD45G expression than the positive group (Figure 6C, right). Overall, these results based on clinical HCC samples showed that GADD45G protein levels correlated with the abundance of SIP1 protein, indicating that GADD45G has a major role in controlling SIP1 expression in human HCC.

## DISCUSSION

Stress sensors GADD45 family proteins have been implicated in the integration of cellular stress signals that regulates cell survival, proliferation and differentiation. GADD45G functions as a tumor suppressive gene via activating an array of downstream effectors to induce cell growth arrest. Particularly, GADD45G can strongly elicit cellular senescence in HCC cells independently of the function of p53, p16 and Rb. In this study, our observations show that SIP1 induction is essential for the stress sensor GADD45G-mediated tumor cell senescence, and that the GADD45G-SIP1 axis is downregulated in clinical HCC.

SIP1, a member of the δ-crystallin enhancer binding factor 1 family, is initially recognized as a two-handed zinc finger transcription factor that binds to Smad1, which plays a critical role in TGF-β signaling and the bone morphogenetic protein (BMP) pathway [32, 33]. The tumor suppressive activity of SIP1 has been appreciated by several studies, in which SIP1 has been shown to induce G1 phase cell cycle arrest by inhibiting cyclin D1 expression in squamous carcinoma cells, or to repress hTERT expression, resulting in replicative senescence in HCC cells [25, 34]. In addition, SIP1 has been found to be downregulated in HCC because of aberrant promoter methylation [35]. In accordance with previous studies, our studies also show that the knockdown of SIP1 restores hTERT expression and contributes to the bypass of cell senescence induced by GADD45G. The present study provides evidence that GADD45G is located upstream of SIP1 in the induction of cellular senescence. It is conceivable that the stress signaling elicited either by environmental clues or by certain agents may affect GADD45G-SIP1 pathway and thereby determine the ultimate fate of the premalignant liver cells or the progression of HCC.

In the present study, we show that JNK activation is the downstream effector of GADD45G that induces SIP1 expression. It has been well acknowledged that JNK is promptly phosphorylated and activated in cellular response to many environmental stresses [36]. JNK activation controls diverse cellular functions such as cell proliferation, aging and apoptosis [37]. For instance, JNK phosphorylates its downstream FOXO4, leading to upregulation of MnSOD expression and EPC senescence [38]. Similarly, PAB triggers a ROS-JNK-p53 positive feedback loop that is a key event in PAB-induced senescence [39]. In line with our observation that ectopic GADD45G expression induces cell senescence and activates the p38 MAPK and JNK pathways, GADD45 proteins have been shown to induce cell cycle arrest at G2/M phase in HepG2 hepatoma cells, wherein the p38 MAPK and JNK act critically [40]. Of note, our results further delineate the distinct roles of p38 MAPK and JNK activation in the GADD45G-induced SIP1 expression and cellular senescence, since the p38 MAPK inhibitor SB203580 suppressed the GADD45G-induced cell senescence but did not change the levels of SIP1. Therefore, p38 MAPK activation and SIP1 induction are two independent events downstream of GADD45G, but, they are functionally integrated in the process of the GADD45G-induced cellular senescence.

We have also reported that GADD45G induces cell senescence by inhibiting Jak/Stat3 activation in HCC cells [23]. Our studies demonstrated that both Stat3 dephosphorylation and SIP1 upregulation contributed to GADD45G-mediated repression of hTERT. With the approaches of Stat3 reactivation by a constitutively active Stat3 mutant and SIP1 knockdown, however, we did not find a mutual regulation between Stat3 signaling and SIP1 expression in the GADD45G-induced cell senescence (data not shown). Strikingly, either Stat3 reactivation or SIP1 inhibition can efficiently counteract GADD45G-induced cell senescence, indicating that Stat3 inhibition and SIP1 activation are parallel, but both of them are essential in GADD45G-induced cell senescence.

Our study demonstrates that decreased GADD45G expression correlated with SIP1 downregultion in clinical HCC tissues, indicating a pathological relevance of the GADD45G-SIP1 axis in HCC development. Considering the efficient activity of GADD45G in triggering HCC cell senescence and suppressing tumor growth, pharmacological activation of GADD45G-SIP1 pathway may have a therapeutic potential for HCC patients, especially for those with the defects in p53/p16 or Rb pathways.

## MATERIALS AND METHODS

### Reagents

The doxycycline (DOX) and puromycin used in this study were purchased from Sigma-Aldrich (St. Louis, MO). MG132, JNK Inhibitor II and p38 inhibitor (SB203580) were purchased from Calbiochem-Novabiochem (San Diego, CA).

### Cell culture

The human liver adenocarcinoma cell line Sk-Hep1 was obtained from the American Type Culture Collection (ATCC, Manassas, VA, USA). The human HCC cell line SMMC-7721 and HEK293T were acquired from the Chinese Academy of Sciences (Shanghai, China). Mouse fetal liver progenitor cells (LPCs) were isolated from p53^−/−^ mice as described previously [41, 42] and cultured for the retroviral delivery of H-Ras V12. All of the cell lines were cultured at 37°C with 5% CO_2_ in Dulbecco's modified Eagle medium supplemented with 10% fetal bovine serum (FBS) and 1% penicillin/streptomycin.

### Lentivirus infection and RNA interference

To establish cell lines with inducible expression of GADD45G, FLAG-tagged human GADD45G cDNA was cloned into pTRIPZ lentiviral vectors (a Tet-on expression vector, Open Biosystems, USA). To produce lentiviral particles, plasmids were transfected into HEK293T packaging cells by using Lipofectamine 2000 reagent (Invitrogen, Carlsbad, CA, USA) according to the manufacturer's protocol. The stable GADD45G-overexpressing cells were selected with 5μg/mL of puromycin for 1 week. The small interfering RNA (siRNA) against GADD45G (SASI_Hs01_00036488) was designed and synthesized by Sigma-Aldrich. The siRNA against SIP1 was synthesized at Invitrogen and the target sequence was as follows: 5′-GCAUGUAU-GCAUGUGACUU-3′. Lentivirus-based knockdown of SIP1 is achieved by construction of pPRIME-CMV-dsRed-FF3 containing the SIP1-targeted sequence.

### RNA isolation and real-time PCR

Total RNA were extracted from tumor cell lines using the RNAiso Reagent (TaKaRa, Dalian, China), and cDNA was prepared using primeScript RT Master kit (Takara). The genes of interest were quantitated by quantitative real-time PCR (Applied Biosystems, Foster City, CA) using SYBR Green PCR Master Mix (TaKaRa). Primers used for reactions are as follows: 5′-AAACAAGCCAATCCCAGGAG-3′ and 5′-GGGTTGGCAATACCGTCATC-3′ for SIP1; 5′-CGGAAGAGTGTCTGG-AGCAA-3′ and 5′-GGATGAAGCGGAGTCTGGA-3′ for hTERT; 5′-ATCACAGG-TTGGAGCCCAGTA-3′ and 5′-CTACCCAGGCATGATCCTCAG-3′ for MEN1; 5′-AGAAAAGACGAGATGGCTCCAG-3′ and 5′-ATCTTCCGATTGCTCCAAA-GAA-3′ for FRK; 5′-TGGTCATCCAATGGAACAGAAA-3′ and 5′-TGCTCTGG-TAGCCATCTTTGAA-3′ for MCPH1; 5′-TCCTGTTCGACAGTCAGCCGCA-3′ and 5′-ACCAGGCGCCCAATACGACCA-3′ for GAPDH. The mRNA level of specific genes was normalized against human GAPDH.

### Western blot analysis

Cell lysates were suspended in RIPA lysis buffer (Beyotime, Nantong, China) including Protease Inhibitor Cocktail and PhosSTOP Phosphatase Inhibitor Cocktail (Roche, Indianapolis, IN, USA). Protein concentrations were determined by BCA assay. Equal amounts of proteins (100μg) were separated on 10% or 12% SDS-PAGE, then transferred to nitrocellulose membranes (Pall, Glen Cove, NY, USA). After being blocked with 5% BSA for 1 hour, the filters were incubated with the primary antibodies at 4°C overnight and incubation with the secondary antibody for 1.5 hours, and the bound was detected with the ChemiDoc^TM^ XRS system (Bio-Rad, Hercules, CA, USA). The antibodies used are as follows: anti-SIP1, anti-GADD45G, anti-GAPDH (Santa Cruz Biotechnology, Santa Cruz, CA, USA); anti-phospho-p38 (Thr180/Tyr182), anti-p38, anti-phospho-JNK1/2 (Thr183/185), anti-JNK1/2, anti-phospho-c-Jun (Ser63), anti-c-Jun (Cell Signaling Technology; CST, Danvers, MA,USA); anti-phospho-HSP27 (Ser82) (Upstate, Lake Placid, NY, USA); anti-HSP27 (Epitomics, Burlingame, CA, USA).

### Senescence-associated β-galactosidase activity assay

To detect the senescence ratio of cells after treatment, SA-β-gal activity was determined by using the SA-β-gal kit (Beyotime, Nantong, China) following the manufacturer's instructions. The SA-β-Gal-positive cells were expressed as a percentage of total cells.

### Cell-cycle analysis

Cells were harvested at indicated times, fixed with 70% cold ethanol. Propidium iodide was added to the cells and the samples were then analyzed by FCM (Becton-Dickinson, California, USA).

### Chromatin immunoprecipitation

Chromatin was immunoprecipitationed with an ChIP assay kit (17-371, Millipore, Billerica, MA, USA) according to the manufacturer's instruction. The antibody used in ChIP experiment is anti-c-Jun from Cell Signaling Technology. The immunoprecipitated DNA was analyzed by qPCR. Primer sequences used were: forward: 5′-CGCTTTGTGTTTGTTACTGTTTGG-3′ and reverse: 5′-TTCCCTCATACGGTCAGGAGTTAT-3′.

### Immunohistochemistry staining

Immunohistochemistry was performed on the tumor sections from sacrificed mice with tumor xenografts to determine the expression of Ki67 (4203-1, Epitomics, dilution 1:100). Tissue sections were deparaffinized and treated with citrate buffer (pH6.0) for antigen retrieval. The sections were incubated with primary antibodies at 4°C overnight. After washing, the sections were incubated with peroxidase-conjugated secondary antibody for 1 hour at room temperature. Next, the slides were rinsed with PBS and incubated for 90 seconds with diaminobenzidine (Metal Enhanced DAB substrate Kit, 34065, ThermoScientific, USA). The slides were washed with distilled water and counterstained with hematoxylin for 30 seconds (Beyotime, Shanghai, China), washed twice with distilled water and examined using a brightfield microscope. Human HCC tissue microarrays were commercially from the Xi'an Alena Biotech Company, and this study was conducted under the approval of the Ethics Committee of Shanghai Cancer Institute. GADD45G (NBP1-55835, NOVUS Biologicals, dilution 1:150) and SIP1 (ab25837, Abcam, dilution 1:150) staining were reported separately according to the German semiquantitative scoring system. In brief, the percentage of the stained cells was classified as follows: 0 (0%), 1 (1-25%), 2 (26-50%), 3 (51-75%), and 4 (76-100%); the staining intensity was graded relatively as : 0 (negative), 1 (weak), 2 (moderate), and 3 (strong). The final immunoreactivity scores were obtained by the formula: IHC scores=percentage score × intensity score.

### Tumor xenograft model

A total of 8×10^6^ viable cells were resuspended in equal volumes of PBS and injected subcutaneously in male BALB/c nude mice (6-8 weeks of age). The mice bearing Tet-on-GADD45G-Sk-Hep1/Vector or Tet-on-GADD45G-Sk-Hep1/shSIP1 were randomly divided into two groups (five mice in each group). One group was given with 2 mg/mL DOX in the drinking water and the other was given normal water. The tumor volumes were monitored by bidimensional measurements using a caliper. Tumor-bearing mice were killed at 50 days after inoculation, and then the tumors were removed for further study. All the animal protocols in this study were approved by the Ethics Committee of Shanghai Cancer Institute.

### Statistical analysis

Statistical analyses were performed using SPSS 16.0. For the *in vivo* studies, the percentage of mice tumor-free in each group was analyzed using a Kaplan-Meier's survival analysis. For the *in vitro* studies, two-tailed *t* test was used to compare the significance between the control and experimental groups. Immunostaining grades were assessed by using Mann-Whitney U test. All of the data represent the mean±SD of three independent experiments. *P* value below 0.05 was considered statistically significant.

## Supplementary MATERIAL TABLE


