# Supplementary material for: SIP1 is a downstream effector of GADD45G in senescence induction and growth inhibition of liver tumor cells
Source: Oncotarget. 2015 Sep 10;6(32):33636–47. doi: 10.18632/oncotarget.5602 (PMC4741791; doi:10.18632/oncotarget.5602)
Supplement: Supplementary file 1 [file oncotarget-06-33636-s001.pdf]

SIP1 is a downstream effector of GADD45G in senescence induction and growth inhibition of liver tumor cells

Supplementary Material

Table 1. Clinicopathological Variables and GADD45G and SIP1 expression in HCC

Patients characteristics	Average (±SD)	Case, n	GADD45G expression			SIP1 expression		
			positive(4-12)	negative(0-3)	*P* value	positive(4-12)	negative(0-3)	*P* value
**Sex**								
Female		11	4	7		5	6	
Male		29	9	20	0.748	14	15	0.873
**Age (yr)**								
＜ 50	43.4±5.3	24	8	16		13	11	
⩾50	58.6±7.0	16	5	11	0.890	6	10	0.301
**Histological grade (Differentation)**								
Well		3	1	2		3	0	
Moderate		30	11	19		17	13	
Poor		7	1	6	0.522	3	4	0.242
Total		40						

P value was studied by chi-square test.
